# Retraction statement: Clinical Characteristics and Risk Factors Associated with Severe Disease Progression Among COVID‐19 Patients in Wad Medani Isolation Centers: A Multicenter Retrospective Cross‐Sectional Study

**DOI:** 10.1002/hsr2.1528

**Published:** 2023-08-24

## Abstract

Mohammed Yousif Elnaeem Yousif, Mohammed Mahmmoud Fadel Allah Eljack, Mazin S. Haroun, Khabab Abbasher Hussien Mohamed Ahmed, Osman Amir, Mohammed Alfatih, Akram Khalid Al Tigany Al Shiekh, Mazin Abdelraham Osman Ahmed, Alshareef Nour, Radi Tofaha Alhusseini, Waddah Aljaely Mohammed Osman, Mohamed Abdulkarim, Mohammed Eltahier Abdalla Omer, Ibrahim M. Mahgoub, HSR2523, https://doi.org/10.1002/hsr2.523.

The above article, published online on {First published: 28 February 2022} in Wiley Online Library {https://onlinelibrary.wiley.com/doi/full/10.1002/hsr2.523} has been retracted by agreement between the journal's Editor‐in‐Chief, Charles Young and John Wiley & Sons Ltd. The retraction has been agreed given the journal has received evidence confirming that the peer review process of this paper was manipulated. As a result, the conclusions reported in the article are not considered reliable.

Mohammed Yousif Elnaeem Yousif, Mohammed Mahmmoud Fadel Allah Eljack, Mazin S. Haroun, Khabab Abbasher Hussien Mohamed Ahmed, Osman Amir, Mohammed Alfatih, Akram Khalid Al Tigany Al Shiekh, Mazin Abdelraham Osman Ahmed, Alshareef Nour, Radi Tofaha Alhusseini, Waddah Aljaely Mohammed Osman, Mohamed Abdulkarim, Mohammed Eltahier Abdalla Omer, Ibrahim M. Mahgoub, HSR2523, https://doi.org/10.1002/hsr2.523.

The above article, published online on {First published: 28 February 2022} in Wiley Online Library {https://onlinelibrary.wiley.com/doi/full/10.1002/hsr2.523} has been retracted by agreement between the journal's Editor‐in‐Chief, Charles Young and John Wiley & Sons Ltd. The retraction has been agreed given the journal has received evidence confirming that the peer review process of this paper was manipulated. As a result, the conclusions reported in the article are not considered reliable.

